# Crystal structures of salts of bedaquiline

**DOI:** 10.1107/S2053229620013455

**Published:** 2020-10-19

**Authors:** Mercy Okezue, Daniel Smith, Matthias Zeller, Stephen R. Byrn, Pamela Smith, Susan Bogandowich-Knipp, Dale K. Purcell, Kari L. Clase

**Affiliations:** a Purdue University, Industrial and Physical Pharmacy, 575 Stadium Mall, West Lafayette, IN 47907, USA; b Purdue University, Chemistry, 560 Oval Drive, West Lafayette, IN 47907-2084, USA; c Improved Pharma, LLC, 1281 Win Hentschel Boulevard, West Lafayette, IN 47906, USA; d Leading with Smart Science, LLC, 5315 Shootingstar Lane, West Lafayette, IN 47906, USA; e Ravine Pharmaceuticals, LLC, 3425 DuBois Street, West Lafayette, IN 47906, USA; f Chemical Microscopy, LLC, 1281 Win Hentschel Boulevard, West Lafayette, IN 47906, USA; g Department of Agricultural & Biological Engineering, Biotechnology Innovation and Regulatory Science Center, Lilly Hall of Life Sciences, Purdue University, 915 State Street, West Lafayette, IN 47906, USA

**Keywords:** bedaquiline, bedaqulinium salt, drug-resistant tuberculosis, crystal structure

## Abstract

Bedaquiline [systematic name: 1-(6-bromo-2-meth­oxy­quinolin-3-yl)-4-(di­methyl­amino)-2-(naphthalen-1-yl)-1-phenyl­butan-2-ol, C_32_H_31_BrN_2_O_2_] is one of two important new drugs for the treatment of drug-resistant tuberculosis (TB). It is marketed in the US as its fumarate salt {systematic name: [4-(6-bromo-2-meth­oxy­quinolin-3-yl)-3-hy­droxy-3-(naphthalen-1-yl)-4-phenyl­but­yl]di­methyl­aza­nium 3-carb­oxy­prop-2-enoate, C_32_H_32_BrN_2_O_2_^+^·C_4_H_3_O_4_^−^}, and about a dozen other salts of bedaquiline have been described in patent literature, but none have so far been structurally described. In a first communication, we present the crystal structure of bedaquilinium fumarate and of two new benzoate salts, as well as that of a degradation product of the reaction of bedaquilinium fumarate with sodium ethoxide, 3-benzyl-6-bromo-2-meth­oxy­quinoline, C_17_H_14_BrNO. The fumarate and benzoate salts both feature cations monoprotonated at the di­methyl­amino group. The much less basic quinoline N atom remains unprotonated. Both salts feature a 1:1 cation-to-anion ratio, with the fumarate being present as monoanionic hydro­fumarate. The conformations of the cations are com­pared to that of free base bedaquiline and with each other. The flexible backbone of the bedaquiline structure leads to a landscape of conformations with little commonalities between the bedaquiline entities in the various structures. The conformations are distinctively different for the two independent mol­ecules of the free base, the two independent mol­ecules of the hydro­fumarate salt, and the one unique cation of the benzoate salt. Packing of the salts is dominated by hydrogen bonding. Hydrogen-bonding motifs, as well as the larger hydrogen-bonded entities within the salts, are quite similar for the salts, despite the vastly differing conformations of the cations, and both the hydro­fumarate and the benzoate structure feature chains of hydrogen-bonded anions that are surrounded by and hydrogen bonded to the larger bedaquilinium cations, leading to infinite broad ribbons of anions, cations, and (for the benzoate salt) water mol­ecules. The benzoate salt was isolated in two forms: as a 1.17-hydrate (C_32_H_32_BrN_2_O_2_^+^·C_7_H_5_O_2_^−^·1.166H_2_O), obtained from acetone or propanol solution, with one fully occupied water mol­ecule tightly integrated into the hydrogen-bonding network of anions and cations, and one partially occupied water mol­ecule [refined occupancy 16.6 (7)%], only loosely hydrogen bonded to the quinoline N atom. The second form is an aceto­nitrile solvate (C_32_H_32_BrN_2_O_2_^+^·C_7_H_5_O_2_^−^·0.742CH_3_CN·H_2_O), in which the partially occupied water mol­ecule is replaced by a 74.2 (7)%-occupied aceto­nitrile mol­ecule. The partial occupancy induces disorder for the benzoate phenyl ring. The aceto­nitrile solvate is unstable in atmosphere and converts into a form not distinguishable by powder XRD from the 1.17-hydrate.

## Abstract 2

The single-crystal structures of three salts of bedaquiline, a drug used for the treatment of drug-resistant tuberculosis (TB), are described.

## Introduction   

Bedaquiline, **1**, is one of two important new drugs for the treatment of drug-resistant tuberculosis (TB). Bedaquiline is marketed in the US as the fumarate salt (**2**) with the trade name Sirturo (Brigden *et al.*, 2015 ▸). The fumarate salt is described in US Patent 8 546 428 (Hegyi *et al.*, 2013 ▸). The citrate, sulfate, phosphate, and tartrate salts are described in two other patents (Zvatora, Dammer, Krejcik *et al.*, 2016 ▸; Zvatora, Dammer, Ridvan *et al.*, 2016 ▸). However, none of these salts has been structurally described in detail. For the fumarate, as well as one each of the two sulfate and citrate salts, well-resolved powder X-ray patterns have been reported, but the structures were not solved and no single-crystal data are reported. For the remaining salts (the phosphate and tartrate salts, and the second sulfate and citrate polymorphs), the powder patterns indicate the samples to have either extremely small particle distributions or to be entirely amorphous. Detailed structural data are reported solely for the free base form of bedaquiline (Petit *et al.*, 2007 ▸).
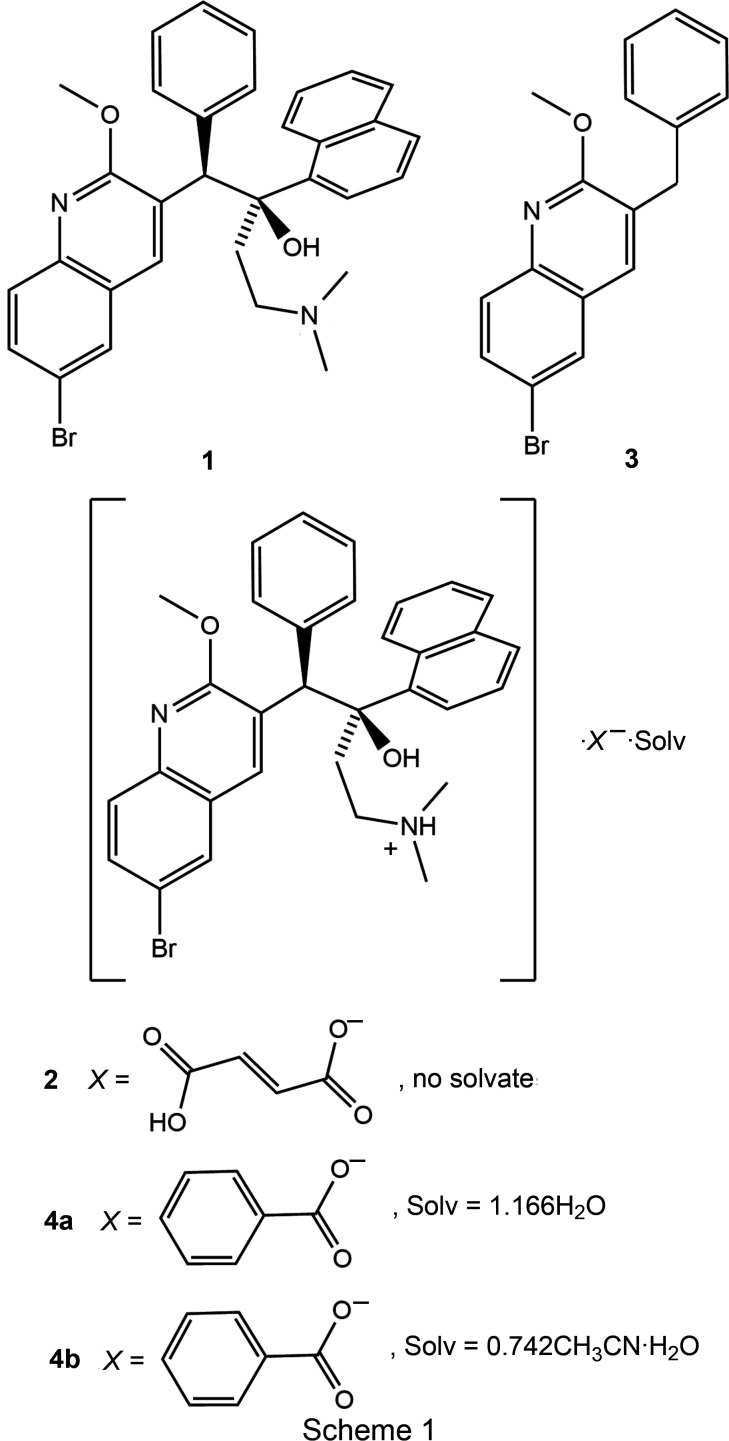


Bedaquiline features two basic N atoms that are amenable to protonation, *i.e.* the tertiary amine appended to the dangling ethyl­ene group and the pyridine N atom. The two sites have distinctly different basicities and selective proton­ation of only the more basic amine site should be possible. Salts of both mono- and dicationic bedaquilinium ions can thus be formed, depending on the strength and amount of acid used for salt formation. The formation of cocrystals (with no or incom­plete proton transfer) can also be imagined.

The NMR data reported in the patent publications indicate bedaquilinium fumarate to have a 1:1 anion-to-cation ratio. Whether the bedaquiline is protonated once or twice (and the fumarate deprotonated once or twice) had not been disclosed. For the sulfate salts, a 1:1 molar ratio of bedaquiline-to-sulfuric acid was used, but the anion-to-cation ratio in the salt was not determined. The given reaction yields, assuming a 1:1 salt, are around 33%, which would allow formation of a 1:2, a 1:1, or a 2:1 salt. The patent specifically states a wide range for the molar ratio of bedaquiline to sulfuric acid: ‘The molar ratio of bedaquiline:sulfuric acid may be in the range of 10:1 to 1:3, preferably 1:1, 2:1, and 1:2’. Similar statements have been made for the tartrate salts, one of the citrate salts, and the phosphate salt. No elemental analysis data are given to support any of the possible ratios, thus leaving the stoi­chi­om­etry and the overall nature of the presented salts in question. The possibility of hydrate or solvate formation was also not properly addressed in the patent claims.

This lack of structural knowledge and even of basic chemical com­position frustrates the understanding of the chemical, physical, and physiological properties of bedaquiline and its derivatives. To reduce this paucity of information on the bedaquiline system, there is inter­est in developing additional salts of bedaquiline and obtaining detailed analysis and structural data for these com­pounds, to better understand and possibly improve their properties, such as solubility, which in turn affect pharmacokinetics and dosage. Additional reasons for this study include finding a bedaquiline salt with improved stability and hygroscopicity.

## Experimental   

Melting points were determined using a Thomas Hoover Capillary Melting Point apparatus and are uncorrected. NMR data were collected in aceto­nitrile-*d*_3_ (ACN-*d*_3_) using a Bruker DRX-500 spectrometer and were referenced against the residual nondeuterated solvent peak.

Benzoic acid was purchased from Mallinckrodt, acetone from Fischer Chemicals, and aceto­nitrile from VWR Chemicals. Hydro­chloride in methanol 1.25 *M* was obtained from Fluka. Bedaquiline fumarate was obtained from Johnson & Johnson. All chemicals were used as received without further purification.

Polarized light microscopy images were collected using an Olympus Series BX51TRF (Olympus America Inc., Melville, NY) polarized light microscope equipped with 12 V/100 W illumination; an Achromat 0.9 NA polarized-light condenser; Olympus Series UPlanFL N objectives: 40X/0.75 NA, 20X/0.50 NA, 10X/0.30 NA, and 4X/0.13 NA; an inter­mediate tube with variable position analyzer and com­pensator; and a tri­nocular viewing head with a Lumenera Series Infinity X (Teledyne Lumenera, Ottawa, Ontario, Canada) digital camera using *Infinity* (Version 6.5.6) and *Infinity Analyze* software (Version 7.0.2.930, Build date 01-Feb-2020). A small portion of sample was placed on a cleaned microscope slide and a No. 1 1/2-cover glass placed over the sample. Mineral oil, USP (CAS: 8042-47-5), was allowed to cover the sample by capillarity. Images were acquired as a collection of three: (i) plane polarized light, (ii) crossed polarized light, and (iii) crossed polarized light with a first-order red com­pensator. Microscopy observations revealed crystal habits for bedaquilinium benzoate powders as birefringent platy anhedral agglomerates that are softly bound and easily dispersed under light pressure from a tungsten needle on the cover glass. A representative collection of images is given in the supporting information.

IR microspectroscopy experiments were conducted using a Smiths Detection (Danbury, CT) IlluminatIR 1.5 IR Microspectrometer accessory on an Olympus Series BX41TF polarized-light microscope (Olympus America Inc., Melville, NY), which provided the base optical platform. The IlluminatIR 1.5 is equipped with a gray-body ceramic IR source, a 60° Michelson Inter­ferometer with a zinc–selenide (ZnSe) beam splitter, a 4 wavenumber (cm^−1^) spectral resolution, and a 0.25 × 0.25 mm liquid-nitro­gen-cooled mercury cadmium telluride (MCT) photoconductive detector, and the sample area was defined using a fixed circular 100 µm aperture. The IlluminatIR 1.5 is com­puter-inter­faced using universal serial bus (USB) communications with Smiths Detection *QualID* App (Version 2.51, 2005) software. Advanced data processing was conducted using either Thermo Galactic spectral analysis software packages *GRAMS/AI* and *SpectralID*, or Thermo Fisher Scientific *OMNIC* software (Version 9.11.706, 2020). IR analyses were performed by reflection/absorption (R/A) using an all-reflecting objective (ARO, 15X, 0.88 NA). A small amount of sample was transferred to a low-E microscope slide (Smiths Detection P/N: 006-4013) and dispersed to a thin layer. IR microprobe analyses were conducted on what appeared microscopically to be a single crystal. An FT–IR spectral background was collected immediately prior to each sample spectral analysis.

Powder X-ray diffraction (XRD) data were collected in focusing mode on a PANalytical Empyrean X-ray diffrac­tometer equipped with Bragg–Brentano HD optics, a sealed-tube copper X-ray source (λ = 1.54178 Å), Soller slits on both the incident and receiving optics sides, and a PixCel3D Medipix detector. Samples were hand ground using an agate mortar and pestle, and packed into a silicon single-crystal zero-background sample holder, 16 mm wide and 0.25 mm deep. Anti­scatter slits and divergence slits, as well as the mask, were chosen based on sample area and starting θ angle. Data were collected between 4 and 40° in 2θ under ambient conditions using the PANalytical *Data Collector* software (PANalytical, 2015 ▸). Rietveld refinements were performed against the 150 K models of the single-crystal structure data sets using *HighScore* (PANalytical, 2015 ▸) software. Refinement of preferred orientation was included using a spherical harmonics model. Plots of Rietveld fits for all com­pounds are given in the supporting information.

### Synthesis and crystallization   

#### Free base bedaquiline (1)   

The free base used during synthesis was prepared by extracting a CH_2_Cl_2_ solution of the fumarate three times with saturated NaHCO_3_ solution (Rombouts *et al.*, 2016 ▸). The identity and purity of the free base thus afforded from the material supplied by Johnson & Johnson was verified using NMR spectroscopy [m.p. 175–176 °C; literature value 181°C (Zvatora, Dammer, Ridvan *et al.*, 2016 ▸)]. ^1^H NMR (500 MHz, ACN-*d*_3_): δ 8.82 (*s*, 1H), 8.66 (*d*, *J* = 8.7 Hz, 1H), 8.03 (*s*, 1H), 8.02 (*d*, *J* = 7.3 Hz, 1H), 7.87 (*d*, *J* = 8.0 Hz, 1H), 7.66 (*t*, *J* = 7.7 Hz, 4H), 7.49 (*t*, *J* = 7.7 Hz, 1H), 7.30 (*m*, 3H), 6.87 (*m*, 3H), 5.88 (*s*, 1H), 4.20 (*s*, 3H), 2.52 (*d*, *J* = 14.6 Hz, 1H), 2.01 (*m*, 2H), 1.89 (*m*, 7H).

Single crystals were grown by dissolving bedaquiline (30 mg, 0.054 mmol) in acetone (1 ml) in a 5 ml scintillation vial and the solution was allowed to evaporate slowly to obtain medium-sized plate-shaped crystals of **1**.

#### Decom­position of bedaquiline fumarate by sodium ethoxide   

Sodium ethoxide (1.5 g, 22.0 mmol) was dissolved in EtOH (20 ml). The resulting solution was added to a solution of bedaquiline fumarate (5 g, 7.44 mmol) in ACN/EtOH (50 ml, 1:1 *v*/*v*). After 1 h, water was added slowly and the resulting mixture extracted with EtOAc. The combined organic layers were dried (MgSO_4_) and then concentrated to provide a colorless crystalline material that was found by IR and NMR spectroscopies to not match free base bedaquiline. Individual crystals were identified as 3-benzyl-6-bromo-2-meth­oxy­quinoline (**3**) by single-crystal X-ray diffraction, and no further analyses were performed.

#### Bedaquilinium fumarate (2)   

Bedaquiline (30 mg, 0.054 mmol) was mixed with fumaric acid (6.3 mg, 0.054 mmol) dissolved in acetone (1 ml) in a 10 ml scintillation vial. Propyl alcohol (5 ml) was then added and the mixture was allowed to evaporate slowly to obtain large colorless block-shaped crystals that were analyzed by single-crystal and powder X-ray diffraction. ^1^H NMR (500 MHz, ACN-*d*_3_): δ 8.68 (*d*, *J* = 8.3 Hz, 1H), 8.55 (*s*, 1H), 8.05 (*d*, *J* = 7.2 Hz, 1H), 7.97 (*s*, 1H), 7.88 (*d*, *J* = 7.9 Hz, 1H), 7.66 (*m*, 4H), 7.51 (*t*, *J* = 7.2 Hz, 1H), 7.32 (*m*, 3H), 6.89 (*m*, 3H), 6.32 (*s*, 2H), 5.89 (*s*, 1H), 4.21 (*s*, 3H), 3.02 (*m*, 1H), 2.69 (*m*, 1H), 2.24 (*s*, 7H), 2.09 (*m*, 2H).

#### Bedaquilinium benzoates 4a and 4b   

Bedaquiline (30 mg, 0.054 mmol) was mixed with benzoic acid (6.7 mg, 0.055 mmol). The mixture was dissolved in acetone (2 ml) in a 5 ml scintillation vial and was allowed to evaporate. The 1.17-hydrate **4a** was obtained in the form of colorless rod-shaped crystals (m.p. 127–129 °C). ^1^H NMR (500 MHz, ACN-*d*_3_): δ 8.75 (*d*, *J* = 8.4 Hz, 1H), 8.67 (*s*, 1H), 8.12 (*d*, *J* = 7.1 Hz, 1H), 7.94 (*d*, *J* = 8.0 Hz, 1H), 7.86 (*m*, 3H), 7.75 (*d*, *J* = 8.0 Hz, 2H), 7.56 (*m*, 4H), 7.38 (*m*, 5H), 6.93 (*m*, 3H), 5.95 (*s*, 1H), 4.26 (*s*, 3H), 3.05 (*m*, 1H), 2.96 (*m*, 1H), 2.25 (*m*, 7H), 1.96 (*m*, 2H). An identical material with the same water content was obtained when crystallization was carried out from 2-propanol instead of acetone.

Bedaquiline (30 mg, 0.054 mmol) was mixed with benzoic acid (6.8 mg, 0.056 mmol) in aceto­nitrile (10 ml) and was allowed to evaporate slowly. The aceto­nitrile solvate monohydrate **4b** was obtained in the form of thin colorless needles (m.p. 127–129 °C).

### Refinement   

Crystal data, data collection, and structure refinement details are summarized in Table 1 ▸. The two benzoate salt structures **4a** and **4b** are isomorphous, differing from each other only in the nature of part of the solvent mol­ecules and some slight shifts to other atoms, and they were refined against a common structural model, with the structure of **4b** being solved by isomorphous replacement from that of **4a**. The atom-naming scheme for the published bedaquiline free base structure (Petit *et al.*, 2007 ▸), as deposited in the Cambridge Structural Database (CSD; Groom *et al.*, 2016 ▸; refcode KIDWAW), was used for the remeasured 150 K data of free base bedaquiline **1** and was also adopted for the bedaquilinium cations in the two benzoate salts **4a** and **4b**, and fumarate salt **2**.

For powder X-ray data collection and refinement, see the *Experimental* (§2).

#### H-atom treatment   

C-bound H atoms were added in calculated positions and refined using a riding model. C—H bond distances were constrained to 0.95 Å for aromatic C—H moieties, and to 1.00, 0.99, and 0.98 Å for aliphatic C—H, CH_2_, and CH_3_ moieties, respectively. Alcohol O—H and ammonium N*R*_3_H N—H bond distances were either freely refined (for **2**) or were constrained to 0.84 and 1.00 Å, respectively. Methyl CH_3_ and hy­droxy H atoms were allowed to rotate but not to tip to best fit the experimental electron density. The positions of the carboxyl­ate H atoms (in **2**) were refined freely. The positions of the fully occupied water H atoms were refined freely and O—H distances were restrained to 0.84 (2) Å. The H atoms of the partially occupied water mol­ecule in **4a** were initially refined and O—H and H⋯H distances were restrained to 0.84 (2) and 1.36 (2) Å, respectively, while a damping factor was applied. The position of water atom H6*E* (in **4a**) was further restrained based on hydrogen-bonding considerations, *i.e.* to be hydrogen bonded to the pyridine H atom, with the H⋯N distance restrained to 2.35 (2) Å. In the final refinement cycles, the damping factor was removed and the H atoms were set to ride on the parent O atom. For all structures, the *U*_iso_(H) values were set to a multiple of *U*_eq_(C), being 1.5 for CH_3_ and OH, and 1.2 for C—H, CH_2_, and N—H groups, respectively.

#### Disorder modeling   

In the structure of **4a**, one fully occupied and one partially occupied water mol­ecule are present in the lattice. The occupancy ratio refined to 0.166 (7). In **4b**, the partially occupied water mol­ecule is replaced by an approximately three-quarter-occupied aceto­nitrile mol­ecule. In the absence of the aceto­nitrile mol­ecule, the neighboring benzene ring of the benzoate anion tilts slightly to move towards the void left by the absent solvent mol­ecule. The two disordered benzene moieties were restrained to have similar geometries. The *U^ij^* com­ponents of the anisotropic displacement parameters (ADPs) for disordered atoms closer to each other than 2.0 Å were restrained to be similar. The ADPs of the *ipso* C atoms, which occupy nearly identical positions, were constrained to be identical. Subject to these conditions, the occupancy ratio refined to 0.742 (7):0.258 (7) in favor of the aceto­nitrile mol­ecule being present.

## Salt screening and methods   

Salt screening is a com­plex and challenging endeavor involving potentially millions of experiments. For bases like bedaquiline, these experiments can involve up to 50 commonly used salt formers in various ratios, as well as crystallizations from up to 60 different solvents by varying temperature, concentration, agitation, pH, and other factors. Further mixtures of these solvents can be used. Anti­solvent crystallization using these solvents is also of inter­est and introduces even more variables.

**Figure 1:**
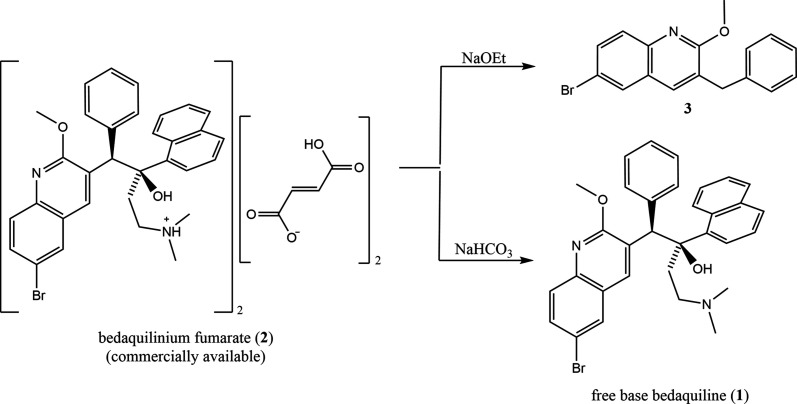
Isolation of free base bedaquiline (**1**) from commercially available bedaquilinium fumarate (**2**), and decom­position to **3**.

For bedaquiline, the first step in screening for additional salts involved recovering bedaquiline free base from the commercially available bedaquilinium fumarate. This was first attempted by deprotonation of the bedaquilinium cation of the fumarate salt using the base sodium ethoxide. However, the alkoxide, when used in excess, proved to be too strong a base and led to fragmentation of the bedaquiline mol­ecule. One of the products of the decom­position reaction was isolated by crystallization and identified, by single-crystal X-ray diffraction, as 3-benzyl-6-bromo-2-meth­oxy­quinoline (**3**) (Fig. 1 ▸) and its structure will be described below. The other half of the decom­position reaction was not recovered or identified, but is assumed to be the ketone elimination product of the remaining bedaquiline fragment, 4-(di­methyl­amino)-1-(naphthalen-1-yl)butan-2-one. The reaction most likely pro­ceeds through initial deprotonation of all acidic groups by the ethoxide, including the central alcohol of bedaquiline. The tertiary alkoxide thus formed can undergo a reverse Grignard reaction (Zook *et al.*, 1959 ▸), under elimination of the ketone and the carbanion of **3**. Using much less basic sodium bicarbonate as the neutralizing agent avoids this decom­position reaction. Bedaquiline free base could be recovered that way from the fumarate salt, following the procedure described by Rombouts *et al.* (2016 ▸), thus allowing us to proceed to use the free base in salt screening experiments (Fig. 1 ▸).

Because the fumarate, citrate, sulfate, phosphate, and tartrate salts were known, salt formation screening focused on the crystallization of salts such as acetate, benzoate, ben­zene­sulfonate, hydro­bromide, succinate (1:1 and 1:2), hydro­chloride, tartrate (1:1 and 1:2), lactate, maleate (1:1 and 1:2), malate (1:1 and 1:2), and mesylate. In general, the crystallizations involved mixing stoichiometric amounts of bedaquiline with the acids at either 1:1 or 1:2 molar ratios in acetone, aceto­nitrile, tetra­hydro­furan, and ethyl acetate, either with or without the anti­solvents water and hexane. The solvents were evaporated either slowly or rapidly, and materials were typically first screened using polarized-light microscopy (PLM) to ensure that a crystalline material had formed, and that the sample was uniform. Samples that passed the first screening step were submitted for further analysis. Crystals were analyzed by NMR (dissolved in an appropriate solvent) to confirm the presence of both com­ponents in the material. In the next step, materials were further screened using IR microspectroscopy, to confirm that the material was indeed a new substance (a salt or a cocrystal), and not just a mixture of the two com­ponents. Although some investigators have advanced the theory that Raman spectroscopy is the best method for analysis and determination of salt formation (*e.g.* Kojima *et al.*, 2010 ▸), we found IR microspectroscopy had better specificity than Raman microscopy for the bedaquiline free base and salts; therefore, screening materials *via* Raman methods was abandoned. IR microspectroscopy proved to be a superior method to determine the formation of bedaquilinium salts. Materials that passed these screening steps (PLM, NMR, and IR spectroscopy) were then analyzed by powder X-ray diffraction. Rietveld refinement was used to identify known crystal phases. For samples for which suitable crystals could be obtained, single-crystal X-ray diffraction was used to determine the structures of phases not yet structurally described.

**Figure 2:**
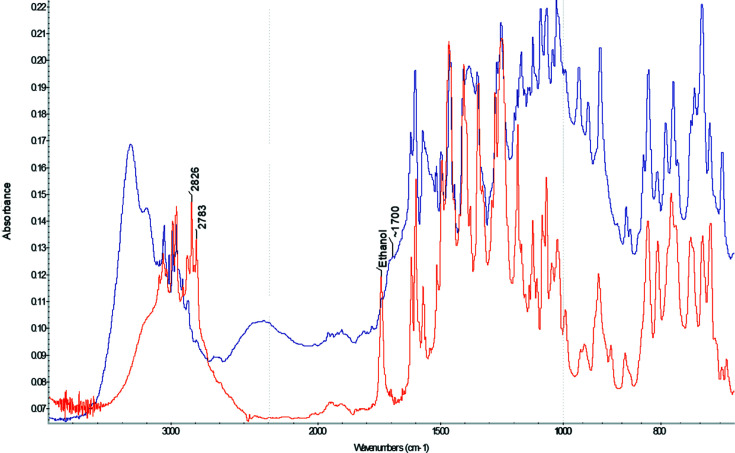
IR spectra of bedaquilinium benzoate (**4a**) (blue) and bedaquiline free base (**1**) (red).

**Figure 3:**
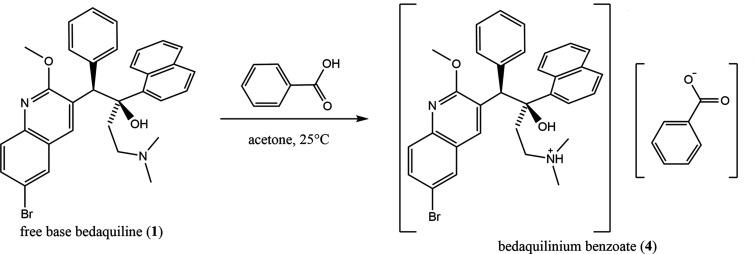
Synthesis of bedaquilinium benzoate (**4**).

Example IR spectra com­paring bedaquilinium benzoate and free base bedaquiline are given in Fig. 2 ▸; see Fig. 3 ▸ for the synthesis. The spectra are distinctly different, indicating transformation of the free base into a material containing both benzoate and bedaquiline fragments. A range of bands in the fingerprint region indicate the presence of a bedaquiline com­ponent in both com­pounds. A shoulder near 1700 cm^−1^ in the bedaquiline benzoate spectrum can be assigned to the C=O stretch of benzoate, confirming the formation of the salt. Further evidence for the formation of a salt, rather than a simple mixture of the two starting materials, is provided by the absence of bands in the range 2830–2760 cm^−1^. Tertiary amines (of which the free base is one) have a characteristic N—CH_2_ in-phase stretch that occurs in this range (Colthup *et al.*, 1990 ▸). The bands in this range of the free base spectrum are not present in the bedaquiline benzoate spectrum, suggesting the formation of a salt. Note: the free base spectrum contains some spectral features due to ethanol, which was used in the synthesis process.

In the course of our investigations, we had been so far able to determine the single-crystal structures of bedaquilinium fumarate (**2**), the commercially available form of bedaquiline, as well as isolate and characterize two other previously unknown bedaquilinium salts: the mono-benzoate salt, in the form of its 1.17-hydrate (**4a**), and a mixed hydrate aceto­nitrile solvate (**4b**). Their structures, as well as that of the degradation product from reaction of bedaquilinium fumarate with sodium ethoxide, 3-benzyl-6-bromo-2-meth­oxy­quinoline (**3**), will be described below. The structure of free base bedaquiline (**1**) was re-recorded at 150 K for easier com­parison with the low-temperature data of **2**, **4a**, and **4b**. Implications for the larger bedaquiline system will be discussed.

## Results and discussion   

**Figure 4:**
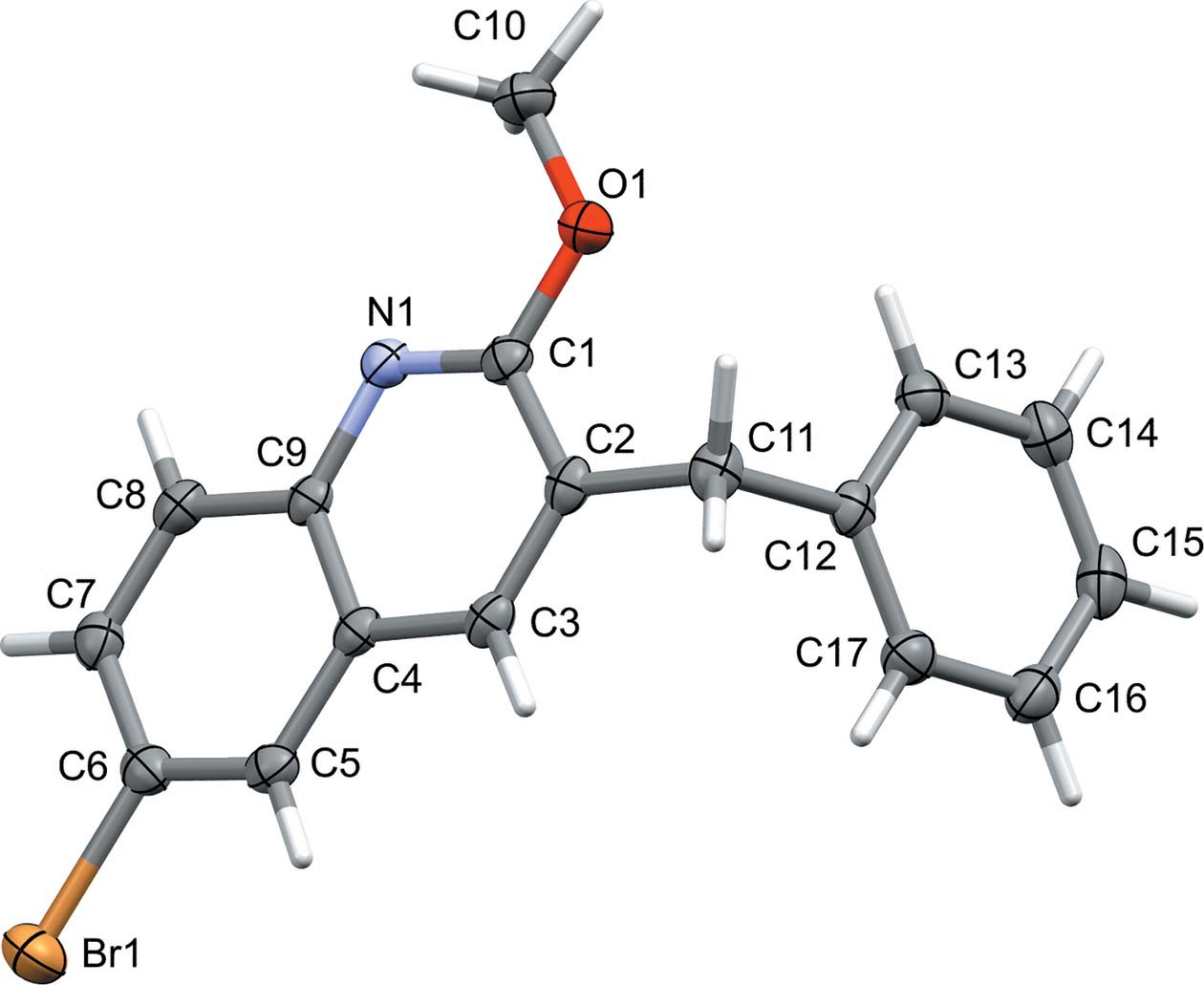
The structure of decom­position product **3** (50% probability displacement ellipsoids).

3-Benzyl-6-bromo-2-meth­oxy­quinoline (**3**), the solvolysis pro­duct from reaction of bedaquiline with sodium ethoxide, lacks an easy-to-identify NMR signature and was identified by single-crystal X-ray diffraction. It crystallized from aceto­nitrile in the ortho­rhom­bic and chiral space group *P*2_1_2_1_2_1_ (Fig. 4 ▸). The starting bedaquilinium fumarate is a chiral com­pound and an enanti­opure sample was used. This chiral information and all chiral centers are, however, lost in the degradation reaction to 3-benzyl-6-bromo-2-meth­oxy­quino­line (**3**). In the solid state, the mol­ecule does, however, exhibit chirality, and the crystal analyzed was found to be enanti­opure, with a Flack parameter of −0.011 (3). In solution, the material is expected to be a rapidly inter­converting racemic mixture, as simple rotation of the benzyl group to the other side of the mean plane of the mol­ecule creates the inversion-related enanti­omer. Mol­ecules of **3** are divided into two planar fragments: the benzyl group and the 6-bromo-2-meth­oxy­quinoline moiety. Both fragments are close to ideally flat, with r.m.s. deviations from planarity of only 0.0052 and 0.0194 Å, respectively. The meth­oxy group is thus ideally coplanar with the remainder of the bromo­quinoline fragment. It points away from the benzyl CH_2_ group to avoid steric inter­actions. The torsion angle between the two mean planes is 73.01 (4)°.

**Figure 5:**
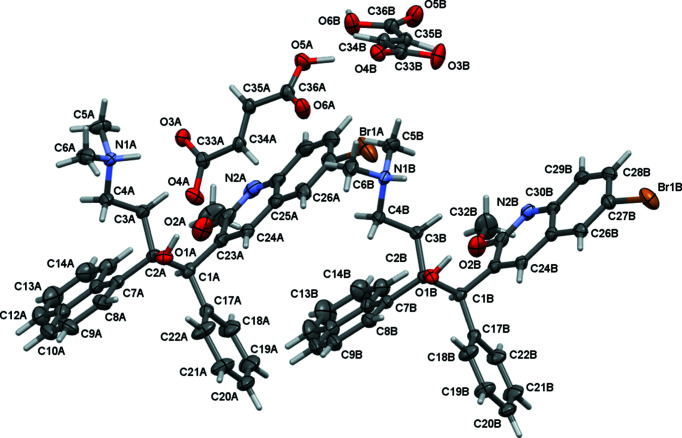
Single-crystal structure of fumarate salt **2** (50% probability displacement ellipsoids).

The structures of the three bedaquilinium salts, *i.e.* fumarate **2**, and benzoates **4a** and **4b**, are substanti­ally more com­plicated (Figs. 5 ▸ and 6 ▸), but they share some commonalities. In all three salt structures, the bedaquilinium cation is singly protonated at the di­methyl­amino fragment, with 1:1 anion-to-cation ratios. In the structure of **2**, the fumarate anions remain singly protonated hydro­fumarate(1−) anions, thus being monoanionic, as are the benzoate anions. The quinoline N atoms remain unprotonated, even though there are additional acidic protons available in the structure of **2**. At first glance, this might be surprising, since many pyridinium salts of both benzoic and fumaric acids have been reported [973 pyridinium benzoate derivatives and 44 pyridinium fumarate salts are reported in the CSD (Groom *et al.*, 2016 ▸), accessed August 2020]. The behaviour is, however, in agreement with the p*K_a_* values of the acids and with the reduced basicity of the quinoline N atom of bedaquiline, com­pared to ordinary pyridine. The first p*K_a_* of fumaric acid is 3.053, the second is 4.494, and that of benzoic acid is 4.202 (Martell & Smith, 1976 ▸), which are easily sufficient to protonate the amine moiety of bedaquiline [the p*K_a_* of tri­alkyl­ammonium ions are around 10–11 (Bioquest, 2020 ▸)]. The p*K_a_* of the conjugated acid of bedaquiline protonated at the quinoline N atom is not reported but can be estimated from the known values for quinoline, pyridine, and 2-meth­oxy­pyridine, which are 4.9, 5.23, and 3.06, respectively (Bioquest, 2020 ▸). Meth­oxy substitution in the 2-position to the N atom substanti­ally reduces the basicity of the N atom (the p*K_a_* of the conjugated acid drops by 2.17 between pyridine and 2-meth­oxy­pyridine). Assuming other effects to be negligible yields a p*K_a_* value of 2.73 for 2-meth­oxy­quinolinium. The quinoline N atom of bedaquiline is thus not basic enough to be protonated by medium-strength acids, such as benzoic or fumaric acid, in agreement with the findings from the crystal structures for **2**, **4a**, and **4b**. Stronger acids, such as mineral acids or maleic acid (first p*K_a_* is 1.94; Bioquest, 2020 ▸), might be able to double protonate bedaquiline if used in sufficient excess. Experiments in this direction are ongoing in our laboratories.

All three salts do crystallize in the chiral monoclinic space group *P*2_1_. The core of the bedaquilinium cation in the three structures is formed by the ethyl­ene moiety of atoms C1 and C2, from which the four major substituents radiate off: the phenyl ring and the bromo­(meth­oxy)quinoline group from C1, and the naphthyl and (di­methyl­amino)­ethyl fragments from C2. The hy­droxy group is also attached to C2, while C1 also carries a single H atom. Atoms C1 and C2 are also the chiral centers of the bedaquilinium cation, which were modeled to have *R* and *S* chiralities, respectively, in agreement with the reported absolute structure for free base bedaquiline (Petit *et al.*, 2007 ▸). The Flack parameters refined to −0.014 (1) for **2**, and to 0.006 (3) and 0.004 (8) for **4a** and **4b**, respectively, confirming that the crystals were enanti­opure.

**Figure 7:**
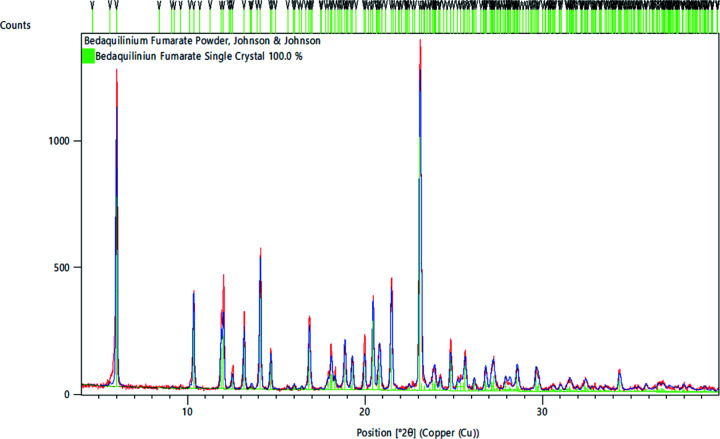
Powder XRD pattern (ambient temperature) of bedaquilinium fumarate (Johnson & Johnson) with a Rietveld refinement fit against the single-crystal structure of **2**. The room-temperature unit-cell parameters refined to *a* = 16.5879 (2), *b* = 10.4952 (8), *c* = 20.183 (2) Å, β = 109.238 (2)° and *V* = 3317.4 (4) Å^3^. Rietveld fits for **4a** and **4b** are given in the supporting information.

The arrangement of anions and cations, and packing inter­actions, however, vary substanti­ally between the fumarate and the two benzoate salts. The fumarate salt crystallized in an anhydrous and unsolvated form. Two crystallographically unique ion pairs are present in the lattice (*i.e. Z*′ = 2 for com­pound **2**). The structure obtained agrees with the reported powder patterns of commercially available bedaquilinium fumarate (see Fig. 7 ▸ for a Rietveld refinement plot).

The two newly isolated benzoate salts are distinctly different from **2**, both being solvates, but they are very similar to each other, and are indeed isomorphous (the aceto­nitrile solvate was solved by isomorphous replacement from the hydrate). Both structures feature one tightly bound water mol­ecule (atom O5). A second inter­stitial site does, however, differ between the two phases. In **4a**, it is occupied by a second water mol­ecule, which is partially occupied. In **4b**, on the other hand, this site is partially occupied by a disordered aceto­nitrile mol­ecule, which in turn induces disorder in the phenyl ring of the benzoate anion [see *Refinement* (§2.2.2) for disorder details].

The ethane backbone of the bedaquiline core gives the cations a three-dimensional (3D) shape, but the individual aromatic fragments remain planar. Similar to **3**, the 6-bromo-2-meth­oxy­quinoline moiety is planar, with the meth­oxy group pointing away from the core of the cation. The r.m.s. deviations from planarity are 0.1127 and 0.1363 for the two cations in **2** (*Z*′ = 2), 0.1019 Å in **4a**, and 0.0922 Å in **4b**.

The ethane backbone and the malleable ethyl­amine fragment gives the bedaquilinium cations a high degree of con­formational flexibility. Differing packing arrangements, induced by the presence (or absence) of varying anions and solvent mol­ecules, led to a landscape of conformations observed for the cations in **2**, **4a**, and **4b**, as well as free base bedaquiline **1**. The dihedral angles between the mean planes of the 6-bromo-2-meth­oxy-quinoline fragment (plane 1), the phenyl ring (plane 2), and the naphthyl group (plane 3), as well as the torsion angles along the ethane backbone and the ethyl­amine fragment, are given in Table 2 ▸. Besides the obvious similarities between the values for isomorphous **4a** and **4b**, no general trend is observed. The conformations vary not only between the four structures, but even between independent mol­ecules within the same structure (both free base **1** and fumarate **2** are *Z*′ = 2 structures). The two C1—C2—C3—C4 torsion angles in fumarate salt **2**, for example, are −63.8 (3) and 174.92 (19)°, which are distinctly different from each other. However, some similarities can be observed: the inter­planar angles between the phenyl and 6-bromo-2-meth­oxy­quinoline planes are between 70 and 90° in all structures, and the torsion angles involving the ethane backbone and the *ipso* phenyl atom (C17—C1—C2—C3) are close to anti­periplanar (‘*trans*’) in all the com­pounds. No other similarities common to all four structures can be found and the overall trend is one of pronounced flexibility and variability.

**Figure 8:**
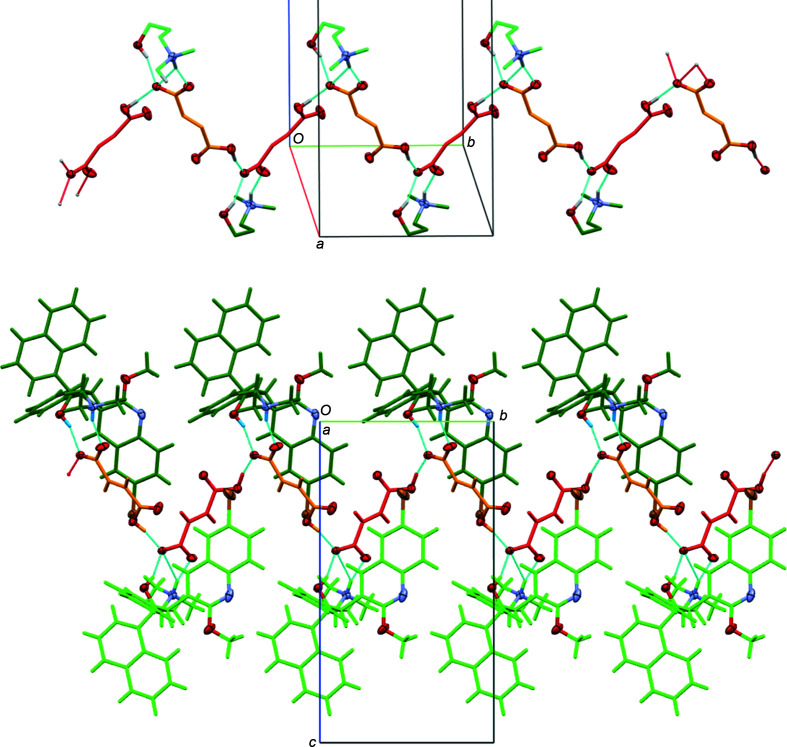
Hydrogen-bonding pattern in **2**. (Top) Chains along *b* created by hydro­fumarate anions. For clarity, only the 3-di­methyl­aza­niumyl-1-hy­droxy­propyl fragments of the cations are shown, and H atoms not involved in hydrogen bonds have been omitted. (Bottom) Segment of an entire ribbon, including full cations. Color coding: O, N, and Br atoms are shown with 50% probability displacement ellipsoids, with all other atoms in capped-stick mode and color coded by the symmetry equivalence of their anion and cation (*A* and *B* cations in light and dark green, and *A* and *B* anions in orange and red). Hydrogen bonds are shown as turquoise and red dashed lines.

While the geometries and conformations in the four bedaquiline structures do not follow any general trend, there are some differences between the geometries of free base bedaquiline and its salts that can be rationalized. Directional inter­actions that differ between the free base and the salts play a major role. In bedaquiline, only one actual hydrogen bond is present, and this is an intra­molecular O—H⋯N hydrogen bond (Table 3 ▸). It induces the amine N atoms to turn towards the hy­droxy group, thus enforcing a *gauche* geometry of the (di­methyl­amino)­ethyl group (see torsion angle C2—C3—C4—N1 in Table 2 ▸). Inter­molecular inter­actions between mol­ecules in **1** are limited to weaker and less directional inter­actions, specifically Br⋯Br inter­actions and π-stacking, which had been discussed in detail by Petit *et al.* (2007 ▸). In the fumarate and benzoate salts **2** and **4**, the opposite is observed. The amine moiety, being protonated, is unable to act as the acceptor for an O—H⋯N hydrogen bond, and it takes up the role of a hydrogen-bond donor towards the benzoate or fumarate anions. This releases the amine from the *gauche* conformation enforced by the intra­molecular O—H⋯N hydrogen bond in **1**, and the ethyl­amine instead extends into a more relaxed conformation, approaching *anti* in **4** and close to eclipsed in **2** (see torsion angle C2—C3—C4—N1 in Table 2 ▸). The hy­droxy groups in the salts are freed up to also form inter­molecular hydrogen bonds. In **4**, they hydrogen bond with the fully occupied water mol­ecule, and in **2** with a fumarate carboxyl­ate group. The water mol­ecule in **4** in turn extends hydrogen bonds to the O atoms of two neighboring benzoate anions, and the fumarate anions in **2** are involved in a series of hydrogen bonds among each other, forming an infinite chain of hydrogen-bonded anions along the *b*-axis direction (Fig. 8 ▸). Thus, one intra­molecular hydrogen bond in free base bedaquiline **1** is converted into a whole series of strong inter­molecular hydrogen bonds (Tables 4 ▸, 5 ▸ and 6 ▸), creating for the salts a much stronger cohesion between neighboring structural entities than what was observed in the free base structure. Weaker inter­actions that dominate for bedaquiline, such as Br⋯Br halogen bonds and π-stacking, are not observed for the salts, but the strong hydrogen bonds are augmented by a range of other not-as-strong directional inter­actions, such as C—H⋯O and C—H⋯Br hydrogen bonds (see hydrogen-bond Tables 4 ▸, 5 ▸, and 6 ▸ for a full listing).

**Figure 9:**
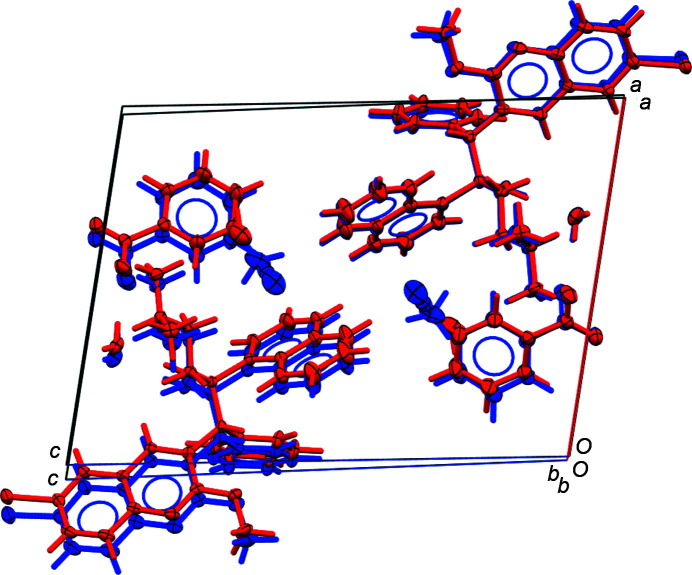
Least-squares overlay of the structures of **4a** (red) and **4b** (blue) using the non-H atoms for one of the two ion pairs of the asymmetric unit (r.m.s. fit value = 0.152), ignoring water and solvent mol­ecules, and disorder. When extending the fit to the atoms of both ion pairs of the unit cell, the r.m.s. value increases to 0.195, showing the lattice mismatch between **4a** and **4b**.

In the aceto­nitrile solvate **4b**, an H atom of the naphthyl group inter­acts in a C—H⋯N hydrogen-like bond with the solvent mol­ecule, when present. In its absence, the phenyl ring of the benzoate anion relaxes towards the void created, inducing disorder for the anion [see *Refinement* (§2.2.2) for details]. For the 1.17-hydrate **4a**, the partially occupied water mol­ecule is hydrogen bonded to the pyridine N atom and acts as an acceptor for a C—H⋯O inter­action. There is, however, no second possible hydrogen-bond acceptor near the partially occupied water mol­ecule, and the second water H atom is not involved in any recognizable inter­action. This is energetically unfavorable, which helps to explain why this position is occupied less than 20% of the time [the refined value is 16.6 (7)%], while the other solvent water mol­ecule, tightly hydrogen bonded on all sides, is fully occupied. Lack of space appears to be no issue, as the larger aceto­nitrile mol­ecule in **4b** has a higher occupancy of around three-quarters [refined value 74.2 (7)%]. Kinetic factors during crystal growth (a lack of water mol­ecules during crystallization from mostly dry solvents for **4a**, but no lack of aceto­nitrile mol­ecules for **4b**) seem to dominate. The presence or absence of either water or aceto­nitrile in this void does not appear to hinder continuation of crystal growth, while this cannot be said for the tightly incorporated O5 water mol­ecule present in both structures, which appears to be essential for the formation of this structure. The larger size of aceto­nitrile *versus* water, and the low prevalence of the second water mol­ecule in **4a**, lead to a slightly larger unit-cell volume for **4b** com­pared to **4a**, *i.e.* 1773.13 (19) *versus* 1729.63 (12) Å^3^. The visually most obvious difference between the two structures is the difference in the β angle, which is expanded by slightly more than 1° in **4b**, leading to a significant offset between atoms in the two structures when the unit cells are overlaid (Fig. 9 ▸). The same is evident when com­paring the powder patterns of **4a** and **4b** simulated from the 150 K single-crystal data, which are distinctively different (see supporting information).

Observations based on powder XRD data indicate that the samples of **4b** quickly desolvate, even under ambient conditions. The powder patterns of **4b** more closely match the parameters of hydrate **4a** than would be expected for aceto­nitrile solvate **4b**, even if the data were collected on samples exposed to air only for a few minutes prior to data collection (Table 7 ▸). Rietveld refinements of samples of **4b** yield β angles that match those of **4a** (at both room temperature and 150 K), and the room-temperature volume of **4b** is actually slightly smaller than that of **4a**, in agreement with the assumption that all the aceto­nitrile in the structure of **4b** is lost when exposed to the atmosphere, while the solvent water mol­ecules of **4a**, being hydrogen bound, do remain in the lattice under these conditions. These observations will be further investigated in a more detailed upcoming study, focusing on a larger series of bedaquilinium salts, including their solvation properties, thermal stability, and responses to ambient moisture.

The structure of **2**, on the other hand, is solvent free. Like **4**, the main inter­molecular inter­actions are strong hydrogen bonds, already briefly discussed, with the bedaquilinium cation always acting as a hydrogen-bond donor, and the hydro­fumarate anions acting as both H-atom acceptors and donors. The quinoline N atom does not act as an acceptor for a hydrogen bond, in agreement with its reduced basicity, already discussed. Originating from the cation are N—H⋯O hydrogen bonds, formed by the ammonium cations, and O—H⋯O hydrogen bonds, originating from the alcohol moieties. The N—H⋯O and O—H⋯O hydrogen bonds from one cation are towards the two O atoms of the same fully deprotonated carboxyl­ate group, yielding an 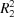
(10) hydrogen-bonding graph-set motif. This motif is identical for the two crystallographically independent ion pairs in **2**. The remaining strong hydrogen bonds are between the hydro­fumarate anions. The hydrogen-bond donors are the carb­oxy­lic acid groups of each hydro­fumarate anion, while the hydrogen-bond acceptor is always the O atom also hydrogen bonded to the bedaquilinium alcohol group, thus tying fumarate anions together in a head-to-tail fashion, forming infinite chains that extend along the *b*-axis direction (Fig. 8 ▸). The individual hydrogen-bonding motifs for these hydrogen bonds are *D*(2). The hydro­fumarate anion chains bridge between bedaquilinium cations connecting anions and cations into ribbons that extend along the *b*-axis direction.

These strong hydrogen bonds in **2** are again augmented by a series of other not-as-strong directional inter­actions, such as C—H⋯O and C—H⋯Br hydrogen bonds (Table 4 ▸) that inter­connect between ribbons to create a fully 3D network and structure. Segments of neighboring ribbons do also inter­digitate with each other, further stabilizing the overall structure.

**Figure 10:**
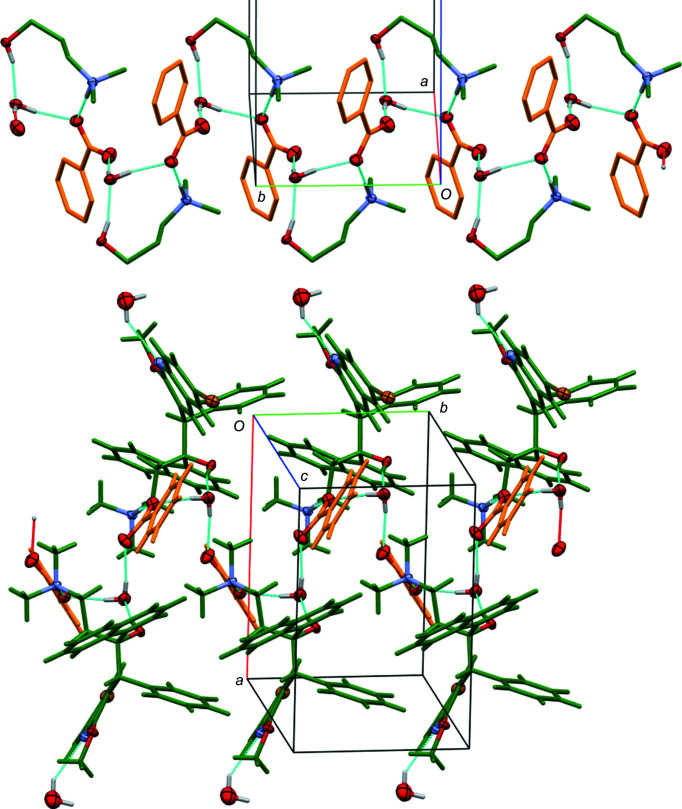
Hydrogen-bonding pattern in **4a**. (Top) Chains along *b* created by benzoate anions and water mol­ecules. For clarity, only the 3-di­methyl­aza­niumyl-1-hy­droxy­propyl fragments of the cations are shown, and H atoms not involved in hydrogen bonds have been omitted. (Bottom) Segment of an entire ribbon, including cations and partially occupied water mol­ecules. Color coding: O, N, and Br atoms are shown with 50% probability displacement ellipsoids, and all other atoms are in capped-stick mode and color coded by the symmetry equivalence of their anion and cation (cations in dark green and benzoate anions in orange). Hydrogen bonds are shown as turquoise and red dashed lines.

The arrangement of anions and cations in **4a** and **4b**, and their connection *via* hydrogen-bonding inter­actions, follows a similar pattern to that in **2**, but it is augmented by the solvate water mol­ecules, which play a similar role as the protonated fumarate carb­oxy­lic acid groups do in **2** in connecting anions and cations with each other into infinite ribbons (Fig. 10 ▸). Bedaquilinium cations and water mol­ecules act as hydrogen-bond donors and the benzoate anions act as hydrogen-bond acceptors. The N—H⋯O hydrogen bond from the cation is towards one O atom of the benzoate carboxyl­ate group and the O—H⋯O hydrogen bond is towards the water mol­ecule, which in turn is hydrogen bonded to the same benzoate O atom as the ammonium fragment. The 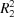
(10) graph-set motif in **2** is thus converted in **4** into an 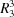
(10) motif, but otherwise fulfils the same function as in **2**, connecting the hy­droxy and ammonium segments of one cation to the same carboxyl­ate group. The fully occupied water mol­ecules in **4** assume the role of the carb­oxy­lic acid groups in **2**, acting as bridges between anions [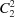
(6) hydrogen-bonding motif], creating infinite benzoate–water chains that extend along the *b*-axis direction. Wrapped around these chains, and connected to them *via* O—H⋯O and N—H⋯O hydrogen bonds, are the bedaquiline cations, thus creating wider ribbons of cations, anions, and solvent water mol­ecules (Fig. 10 ▸), emulating the pattern already observed for **2**. The partially occupied water mol­ecules are located on the outer rim of the ribbon, hydrogen bonded to the quinoline N atom but not bridging or con­necting between any structural entities.

The strong inter­molecular inter­actions present in **4a** and **4b** do com­pensate for the presence of the only partially or not at all filled voids present, and the packing efficiency for the salts of **4** is com­parable to that of free base bedaquiline. Indeed, the density for the 1.17-hydrate is, at 1.342 Mg m^−3^, virtually identical to that of the free base of 1.344 Mg m^−3^ (both measured at 150 K), and the aceto­nitrile solvate is, at 1.360 Mg m^−3^, even denser than the solvent-free base. The best packing efficiency is, however, observed for fumarate salt **2**, featuring a multitude of strong hydrogen bonds, and having neither incorporated solvent mol­ecules nor unoccupied void space. Its density, at 150 K, is 1.379 Mg m^−3^ (Table 7 ▸).

## Supplementary Material

Crystal structure: contains datablock(s) 1, 2, 3, 4a, 4b, global. DOI: 10.1107/S2053229620013455/ov3143sup1.cif

Structure factors: contains datablock(s) 1. DOI: 10.1107/S2053229620013455/ov31431sup2.hkl

Structure factors: contains datablock(s) 2. DOI: 10.1107/S2053229620013455/ov31432sup3.hkl

Structure factors: contains datablock(s) 3. DOI: 10.1107/S2053229620013455/ov31433sup4.hkl

Structure factors: contains datablock(s) 4a. DOI: 10.1107/S2053229620013455/ov31434asup5.hkl

Structure factors: contains datablock(s) 4b. DOI: 10.1107/S2053229620013455/ov31434bsup6.hkl

Supporting information file. DOI: 10.1107/S2053229620013455/ov31431sup7.cml

Supporting information file. DOI: 10.1107/S2053229620013455/ov31432sup8.cml

Supporting information file. DOI: 10.1107/S2053229620013455/ov31433sup9.cml

Supporting information file. DOI: 10.1107/S2053229620013455/ov31434asup10.cml

Supporting information file. DOI: 10.1107/S2053229620013455/ov31434bsup11.cml

Polarized light microscopy images and PXRD parretns. DOI: 10.1107/S2053229620013455/ov3143sup12.pdf

CCDC references: 2036005, 2036004, 2036003, 2036002, 2036001

## Figures and Tables

**Figure 6:**
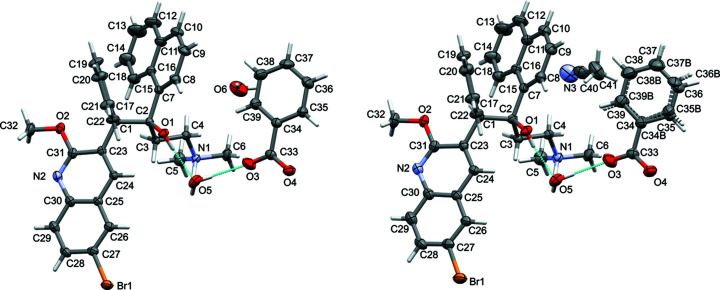
Single-crystal structures of benzoate salts of **4a** (left) and **4b** (right) (50% probability displacement ellipsoids). Hydrogen bonds are shown as turquoise dashed lines.

**Table:** Experiments were carried out at 150 K. Absorption was corrected for by multi-scan methods (*SADABS2016*; Krause *et al.*, 2015 ▸).

	**1**	**2**	**3**
Crystal data			
Chemical formula	C_32_H_31_BrN_2_O_2_	C_32_H_32_BrN_2_O_2_^+^·C_4_H_3_O_4_^−^	C_17_H_14_BrNO
*M*_r_	555.50	671.57	328.20
Crystal system, space group	Orthorhombic, *P*2_1_2_1_2_1_	Monoclinic, *P*2_1_	Orthorhombic, *P*2_1_2_1_2_1_
*a*, *b*, *c* (Å)	11.1584 (8), 13.6425 (14), 36.061 (4)	16.4556 (6), 10.3205 (3), 20.1636 (8)	4.3606 (6), 10.820 (2), 29.886 (11)
α, β, γ (°)	90, 90, 90	90, 109.1832 (15), 90	90, 90, 90
*V* (Å^3^)	5489.5 (9)	3234.2 (2)	1410.1 (6)
*Z*	8	4	4
Radiation type	Mo *K*α	Mo *K*α	Mo *K*α
μ (mm^−1^)	1.53	1.32	2.91
Crystal size (mm)	0.21 × 0.13 × 0.05	0.45 × 0.37 × 0.17	0.41 × 0.06 × 0.05

Data collection			
Diffractometer	Bruker D8 Quest diffractometer with PhotonII charge-integrating pixel array detector (CPAD)	Bruker D8 Quest diffractometer with PhotonII charge-integrating pixel array detector (CPAD)	Bruker D8 Quest diffractometer with PhotonII charge-integrating pixel array detector (CPAD)
*T*_min_, *T*_max_	0.603, 0.747	0.438, 0.495	0.658, 0.747
No. of measured, independent and observed [*I* > 2σ(*I*)] reflections	66520, 17893, 12296	115858, 24622, 18572	28030, 5125, 4504
*R*_int_	0.052	0.040	0.037
(sin θ/λ)_max_ (Å^−1^)	0.770	0.771	0.768

Refinement			
*R*[*F* ^2^ > 2σ(*F* ^2^)], *wR*(*F* ^2^), *S*	0.044, 0.111, 1.03	0.043, 0.117, 1.06	0.023, 0.057, 1.05
No. of reflections	17893	24622	5125
No. of parameters	675	837	196
No. of restraints	0	1	0
H-atom treatment	H-atom parameters constrained	H atoms treated by a mixture of independent and constrained refinement	Only H-atom displacement parameters refined
Δρ_max_, Δρ_min_ (e Å^−3^)	0.48, −0.58	1.23, −1.28	0.28, −0.38
Absolute structure	Flack *x* determined using 4397 quotients [(*I* ^+^) − (*I* ^−^)]/[(*I* ^+^) + (*I* ^−^)] (Parsons *et al.*, 2013 ▸)	Flack *x* determined using 7327 quotients [(*I* ^+^) − (*I* ^−^)]/[(*I* ^+^) + (*I* ^−^)] (Parsons *et al.*, 2013 ▸)	Flack *x* determined using 1685 quotients [(*I* ^+^) − (*I* ^−^)]/[(*I* ^+^) + (*I* ^−^)] (Parsons *et al.*, 2013 ▸)
Absolute structure parameter	0.034 (3)	−0.0144 (14)	−0.011 (3)

**Table:** 

	**4a**	**4b**
Crystal data		
Chemical formula	C_32_H_32_BrN_2_O_2_^+^·C_7_H_5_O_2_^−^·1.166H_2_O	C_32_H_32_BrN_2_O_2_^+^·C_7_H_5_O_2_^−^·0.742C_2_H_3_N·H_2_O
*M*_r_	698.70	726.10
Crystal system, space group	Monoclinic, *P*2_1_	Monoclinic, *P*2_1_
*a*, *b*, *c* (Å)	12.6384 (5), 7.9259 (3), 17.5249 (8)	12.8661 (8), 8.0386 (5), 17.4704 (10)
α, β, γ (°)	90, 99.8450 (17), 90	90, 101.093 (3), 90
*V* (Å^3^)	1729.63 (12)	1773.13 (19)
*Z*	2	2
Radiation type	Mo *K*α	Cu *K*α
μ (mm^−1^)	1.24	1.97
Crystal size (mm)	0.55 × 0.21 × 0.13	0.31 × 0.05 × 0.05

Data collection		
Diffractometer	Bruker D8 Quest diffractometer with PhotonII charge-integrating pixel array detector (CPAD)	Bruker D8 Quest diffractometer with PhotonIII_C14 charge-integrating and photon counting pixel array detector
*T*_min_, *T*_max_	0.638, 0.746	0.599, 0.754
No. of measured, independent and observed [*I* > 2σ(*I*)] reflections	80228, 13080, 10456	39739, 7360, 6750
*R*_int_	0.049	0.060
(sin θ/λ)_max_ (Å^−1^)	0.770	0.639

Refinement		
*R*[*F* ^2^ > 2σ(*F* ^2^)], *wR*(*F* ^2^), *S*	0.032, 0.073, 1.03	0.035, 0.085, 1.06
No. of reflections	13080	7360
No. of parameters	445	515
No. of restraints	5	195
H-atom treatment	H atoms treated by a mixture of independent and constrained refinement	H atoms treated by a mixture of independent and constrained refinement
Δρ_max_, Δρ_min_ (e Å^−3^)	0.36, −0.48	0.40, −0.50
Absolute structure	Flack *x* determined using 4051 quotients [(*I* ^+^) − (*I* ^−^)]/[(*I* ^+^) + (*I* ^−^)] (Parsons *et al.*, 2013 ▸)	Flack *x* determined using 2778 quotients [(*I* ^+^) − (*I* ^−^)]/[(*I* ^+^) + (*I* ^−^)] (Parsons *et al.*, 2013 ▸)
Absolute structure parameter	0.006 (3)	0.004 (8)

Computer programs: *APEX3* (Bruker, 2019 ▸), *SAINT* (Bruker, 2019 ▸), *SHELXS97* (Sheldrick, 2008 ▸), *SHELXL2018* (Sheldrick, 2015 ▸), *shelXle* (Hübschle *et al.*, 2011 ▸), *Mercury* (Macrae *et al.*, 2020 ▸) and *publCIF* (Westrip, 2010 ▸).

**Table 2:** Selected torsion angles (°) for free base bedaquiline **1**, fumarate salt **2**, and benzoate salts **4a** and **4b**

	**1** ^*a*,*b*^	**2** ^*b*^	**4a** ^*a*^	**4b** ^*a*^
τ plane 1 *versus* plane 2^*c*^	73.29 (7), 81.37 (7)	85.33 (9), 86.02 (8)	77.31 (6)	76.2 (1)
τ plane 2 *versus* plane 3^*c*^	86.13 (8) 76.95 (7)	89.7 (1), 89.74 (9)	45.62 (6)	44.2 (1)
τ plane 1 *versus* plane 3^*c*^	14.0 (1), 8.16 (9)	8.71 (3), 16.67 (4)	37.50 (6)	36.7 (1)
τ C1—C2—C3—C4	175.7 (3), 177.0 (3)	−63.8 (3), 174.92 (19)	166.84 (14)	165.1 (3)
τ C2—C3—C4—N1	58.7 (4), 63.0 (4)	137.2 (2), 133.7 (2)	164.04 (15)	165.5 (3)
τ C17—C1—C2—C3	169.0 (3), 178.8 (3)	169.7 (2), 171.38 (19)	174.44 (14)	174.1 (3)

Notes: (*a*) measured at 150 K; (*b*) *Z*′ = 2; (*c*) plane 1 = 6-bromo-2-meth­oxy­quinoline, plane 2 = phenyl, and plane 3 = naphthyl.

**Table 3:** Hydrogen-bond geometry (Å, °) for **1** [Chem scheme1]

*D*—H⋯*A*	*D*—H	H⋯*A*	*D*⋯*A*	*D*—H⋯*A*
O1—H1O⋯N1	0.84	1.94	2.696 (4)	150
C1—H1⋯O2	1.00	2.24	2.763 (4)	111
O3—H3O⋯N3	0.84	1.93	2.685 (4)	149
C33—H33⋯O4	1.00	2.23	2.773 (4)	112

**Table 4:** Hydrogen-bond geometry (Å, °) for **2** [Chem scheme1]

*D*—H⋯*A*	*D*—H	H⋯*A*	*D*⋯*A*	*D*—H⋯*A*
O1*A*—H1*AB*⋯O4*A*	0.86 (4)	1.88 (4)	2.699 (3)	159 (4)
O5*A*—H5*A*⋯O4*B*	1.04 (4)	1.58 (4)	2.603 (3)	169 (4)
N1*A*—H1*A*N⋯O3*A*	0.98 (4)	1.68 (4)	2.641 (3)	163 (3)
C1*A*—H1*A*⋯O2*A*	1.00	2.23	2.767 (3)	112
C3*A*—H3*AB*⋯Br1*B* ^i^	0.99	3.01	3.850 (3)	143
C5*A*—H5*AA*⋯O1*A* ^ii^	0.98	2.58	3.501 (3)	157
C5*A*—H5*AC*⋯Br1*B* ^i^	0.98	2.82	3.612 (3)	139
C26*A*—H26*A*⋯O5*B* ^iii^	0.95	2.54	3.459 (4)	163
C34*A*—H34*A*⋯O5*B* ^iii^	0.95	2.52	3.379 (3)	151
O1*B*—H1*B*⋯O4*B* ^iv^	0.89 (4)	1.88 (4)	2.741 (3)	160 (4)
O6*B*—H6*B*⋯O4*A* ^v^	0.86 (5)	1.80 (5)	2.625 (3)	159 (4)
N1*B*—H1*B*N⋯O3*B* ^iv^	0.85 (4)	1.83 (4)	2.632 (3)	158 (4)
C5*B*—H5*BA*⋯O1*B* ^vi^	0.98	2.42	3.337 (4)	156
C5*B*—H5*BB*⋯Br1*A*	0.98	2.88	3.468 (3)	119
C5*B*—H5*BB*⋯O6*A*	0.98	2.42	3.287 (4)	148

Symmetry codes: (i) 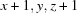; (ii) 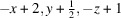; (iii) 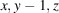; (iv) 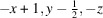; (v) 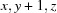; (vi) 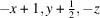.

**Table 5:** Hydrogen-bond geometry (Å, °) for **4a** [Chem scheme1]

*D*—H⋯*A*	*D*—H	H⋯*A*	*D*⋯*A*	*D*—H⋯*A*
O1—H1*B*⋯O5	0.84	1.87	2.681 (2)	164
O5—H5*D*⋯O4^i^	0.90 (4)	1.85 (4)	2.724 (2)	163 (3)
O5—H5*E*⋯O3	0.77 (3)	1.96 (3)	2.724 (2)	172 (4)
O6—H6*E*⋯N2	0.87	2.38	3.148 (13)	147
N1—H1⋯O4^i^	1.00	1.64	2.643 (2)	178
C5—H5*C*⋯Br1^ii^	0.98	3.09	3.910 (2)	142
C6—H6*A*⋯O3	0.98	2.53	3.465 (3)	161
C6—H6*B*⋯O6^iii^	0.98	2.28	3.249 (13)	169
C28—H28⋯O5^ii^	0.95	2.59	3.421 (3)	146

Symmetry codes: (i) 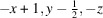; (ii) 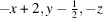; (iii) 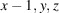.

**Table 6:** Hydrogen-bond geometry (Å, °) for **4b** [Chem scheme1]

*D*—H⋯*A*	*D*—H	H⋯*A*	*D*⋯*A*	*D*—H⋯*A*
O1—H1*B*⋯O5	0.77	1.94	2.691 (4)	163
O5—H5*D*⋯O4^i^	0.86 (3)	1.89 (3)	2.738 (4)	168 (6)
O5—H5*E*⋯O3	0.82 (3)	1.92 (3)	2.734 (4)	172 (6)
N1—H1⋯O4^i^	1.00	1.62	2.619 (4)	178
C5—H5*C*⋯Br1^ii^	0.98	3.13	3.964 (4)	144
C6—H6*A*⋯O3	0.98	2.63	3.562 (6)	159
C28—H28⋯O5^ii^	0.95	2.66	3.498 (5)	148

Symmetry codes: (i) 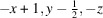; (ii) 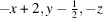.

**Table 7:** 150 K and room-temperature (RT) unit-cell dimensions for **1**, **2**, **4a**, and **4b** RT data were obtained from powder XRD patterns *via* Rietveld refinement (see supporting information for Rietveld plots).

	**1**, 150 K	**1**, RT	**2**, 150 K	**2**, RT	
*a* (Å)	11.1584 (8)	11.230 (1)	16.4556 (6)	16.5879 (2)	
*b* (Å)	13.6425 (14)	13.766 (1)	10.3205 (3)	10.4952 (8)	
*c* (Å)	36.061 (4)	36.455 (3)	20.1636 (8)	20.183 (2)	
β (°)	90	90	109.1832 (15)	109.238 (2)	
*V* (Å^3^)	5489.5 (9)	5636 (1)	3234.2 (2)	3317.4 (4)	
ρ (Mg m^−3^)	1.344	1.309	1.379	1.344	
*R*_wp_ (%)		19.99		20.48	
*R*_exp _ (%)		15.67		13.27	
*S*		1.27		1.54	
					
	**4a**, 150 K	**4a**, RT	**4b**, 150 K	**4b**, RT^*a*^	
*a* (Å)	12.6384 (5)	12.7267 (4)	12.8661 (8)	12.720 (1)	12.7242 (8)
*b* (Å)	7.9259 (3)	8.0157 (3)	8.0386 (5)	8.0094 (5)	8.0116 (6)
*c* (Å)	17.5249 (8)	17.6438 (7)	17.4704 (10)	17.626 (2)	17.632 (1)
β (°)	99.8450 (17)	99.7928 (6)	101.093 (3)	99.836 (2)	99.813 (1)
*V* (Å^3^)	1729.63 (12)	1773.7 (1)	1773.13 (19)	1769.4 (3)	1771.2 (2)
ρ (Mg m^−3^)	1.342	1.309	1.360	1.311	1.361
*R*_wp_ (%)		5.57		30.65	28.65
*R*_exp _ (%)		10.78		10.50	14.26
*S*		1.94		2.12	2.01

Note: (*a*) data from two independent samples and Rietveld refinements.

## References

[bb1] Bioquest (2020). *AAT Bioquest pK_a_ and pK_b_ Reference Table*, https://www.aatbio.com/data-sets/pka-and-pkb-reference-table, accessed August 21, 2020.

[bb2] Brigden, G., Hewison, C. & Varaine, F. (2015). *Infect. Drug Resist.* **8**, 367–378. 10.2147/IDR.S68351 PMC4634826 26586956

[bb3] Bruker (2019). *APEX3* and *SAINT*. Bruker AXS Inc., Madison, Wisconsin, USA.

[bb4] Colthup, N. B., Daly, L. H. & Wiberley, S. E. (1990). In *Introduction to Infrared and Raman Spectroscopy*, 3rd ed. New York: Academic Press Inc.

[bb5] Groom, C. R., Bruno, I. J., Lightfoot, M. P. & Ward, S. C. (2016). *Acta Cryst.* B**72**, 171–179. 10.1107/S2052520616003954 PMC4822653 27048719

[bb6] Hegyi, J. F. A. L., Aelterman, W. A. A., Lang, Y. L., Stokbroekx, S. C. M., Leys, C., Van Remoortere, P. J. M. & Faure, A. (2013). US Patent 8 546 428, Janssen Pharmaceuticals, USA.

[bb7] Hübschle, C. B., Sheldrick, G. M. & Dittrich, B. (2011). *J. Appl. Cryst.* **44**, 1281–1284. 10.1107/S0021889811043202 PMC3246833 22477785

[bb8] Kojima, T., Tsutsumi, S., Yamamoto, K., Ikeda, Y. & Moriwaki, T. (2010). *Int. J. Pharm.* **399**(1–2), 52–59. 10.1016/j.ijpharm.2010.07.055 20696223

[bb9] Krause, L., Herbst-Irmer, R., Sheldrick, G. M. & Stalke, D. (2015). *J. Appl. Cryst.* **48**, 3–10. 10.1107/S1600576714022985 PMC4453166 26089746

[bb10] Macrae, C. F., Sovago, I., Cottrell, S. J., Galek, P. T. A., McCabe, P., Pidcock, E., Platings, M., Shields, G. P., Stevens, J. S., Towler, M. & Wood, P. A. (2020). *J. Appl. Cryst.* **53**, 226–235. 10.1107/S1600576719014092 PMC6998782 32047413

[bb11] Martell, A. E. & Smith, R. M. (1976). In *Critical Stability Constants*, Vols. 1–4. New York: Plenum Press.

[bb12] PANalytical (2015). *Data Collector* (XRD data collection software, Version 5.3.0.62) and *HighScore* (Version 4.5). PANalytical BV, Almelo, The Netherlands.

[bb13] Parsons, S., Flack, H. D. & Wagner, T. (2013). *Acta Cryst.* B**69**, 249–259. 10.1107/S2052519213010014 PMC3661305 23719469

[bb14] Petit, S., Coquerel, G., Meyer, C. & Guillemont, J. (2007). *J. Mol. Struct.* **837**, 252–256.

[bb15] Rombouts, J. A., Veenboer, R. P., Villellas, C., Lu, P., Ehlers, A. W., Andries, K., Koul, A., Lill, H., Ruijter, E., Orru, R. V. A., Lammertsma, K., Bald, D. & Slootweg, J. C. (2016). *RSC Adv.* **6**, 108708–108716.

[bb16] Sheldrick, G. M. (2008). *Acta Cryst.* A**64**, 112–122. 10.1107/S0108767307043930 18156677

[bb17] Sheldrick, G. M. (2015). *Acta Cryst.* C**71**, 3–8.

[bb18] Westrip, S. P. (2010). *J. Appl. Cryst.* **43**, 920–925.

[bb19] Zook, H. D., March, J. & Smith, D. F. (1959). *J. Am. Chem. Soc.* **81**, 1617–1620.

[bb20] Zvatora, P., Dammer, O., Krejcik, L., Zvonicek, V. & Hert, J. (2016). Int. Patent WO/2016/198031A1, Zentiva, Czech Republic.

[bb21] Zvatora, P., Dammer, O., Ridvan, L., Lustig, P., Pekarek, T., Stefco, M., Krejcik, L. & Tkadlecova, M. (2016). Int. Patent WO/2016/058564, Zentiva, Czech Republic.

