# Supplementary material for: Crystal structures of salts of bedaquiline
Source: Acta Crystallogr C Struct Chem. 2020 Oct 19;76(Pt 11):1010–23. doi: 10.1107/S2053229620013455 (PMC7642769; doi:10.1107/S2053229620013455)
Supplement: Supplementary file 12 [file c-76-01010-sup12.pdf]

STRUCTURAL CHEMISTRY

Volume 76 (2020)

Supporting information for article:

Crystal structures of salts of bedaquiline

Mercy Okezue, Daniel Smith, Matthias Zeller, Stephen R. Byrn, Pamela Smith, Susan Bogandowich-Knipp, Dale K. Purcell and Kari L. Clase

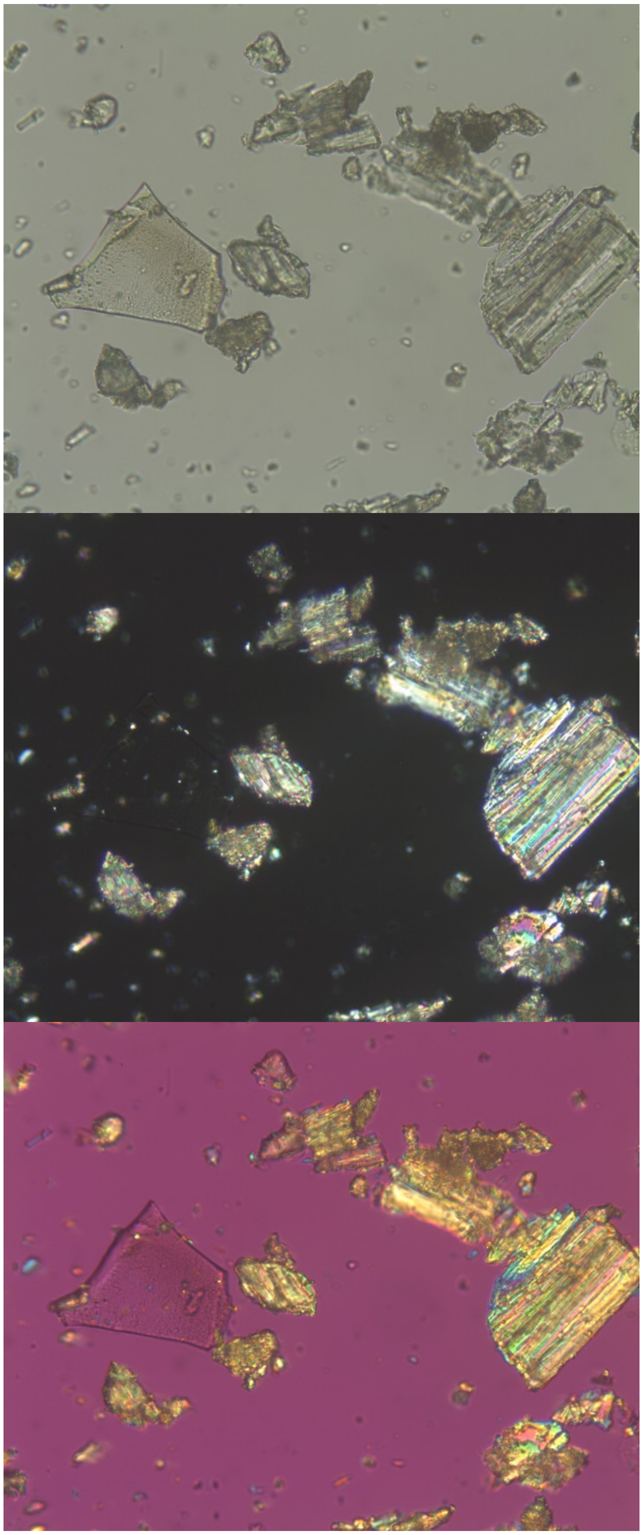

Figure S1: Polarized light microscopy of bedaquiline benzoate with (top to bottom) plane polarized light, crossed polarized light, and crossed polarized light with first-order red compensator

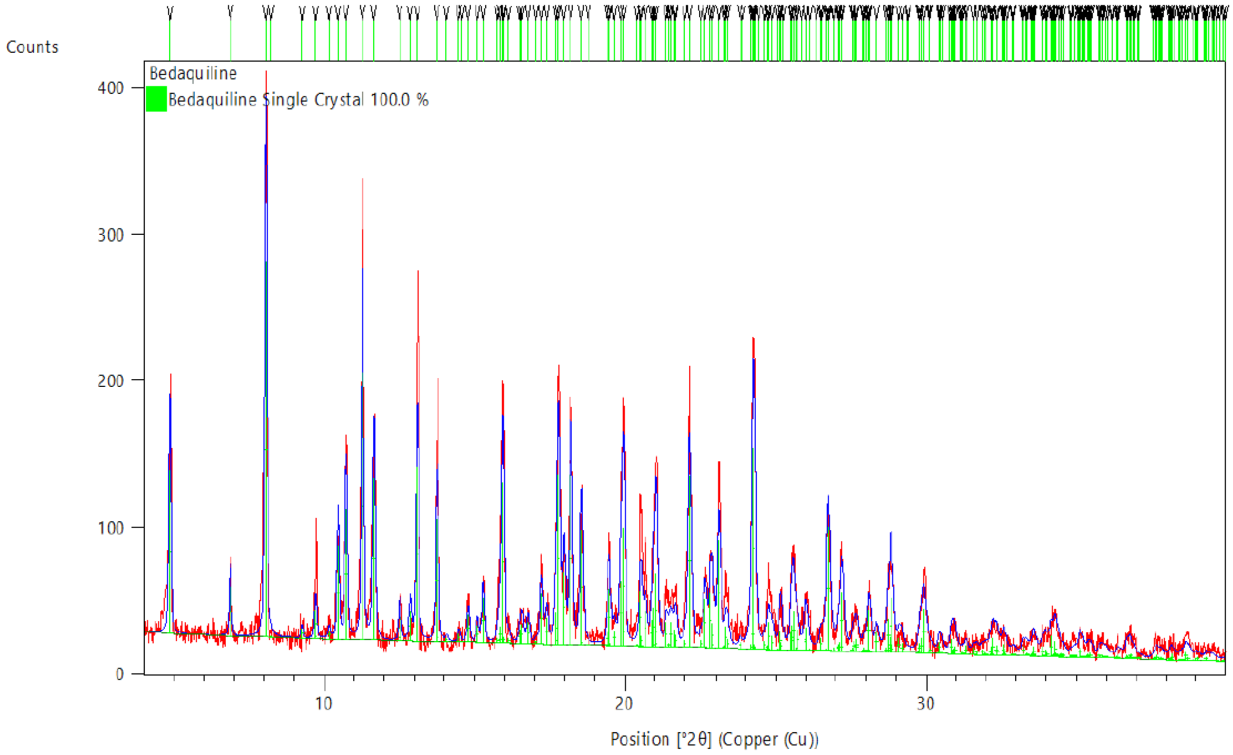

Figure S2. Powder XRD pattern (ambient temperature) of bedaquiline powder with Rietveld refinement fit against the single crystal structure of **1**. The room temperature unit cell parameters refined to a = 11.230(1), b = 13.766(1), c = 36.455(3) Å, V = 5636(1) Å^3.

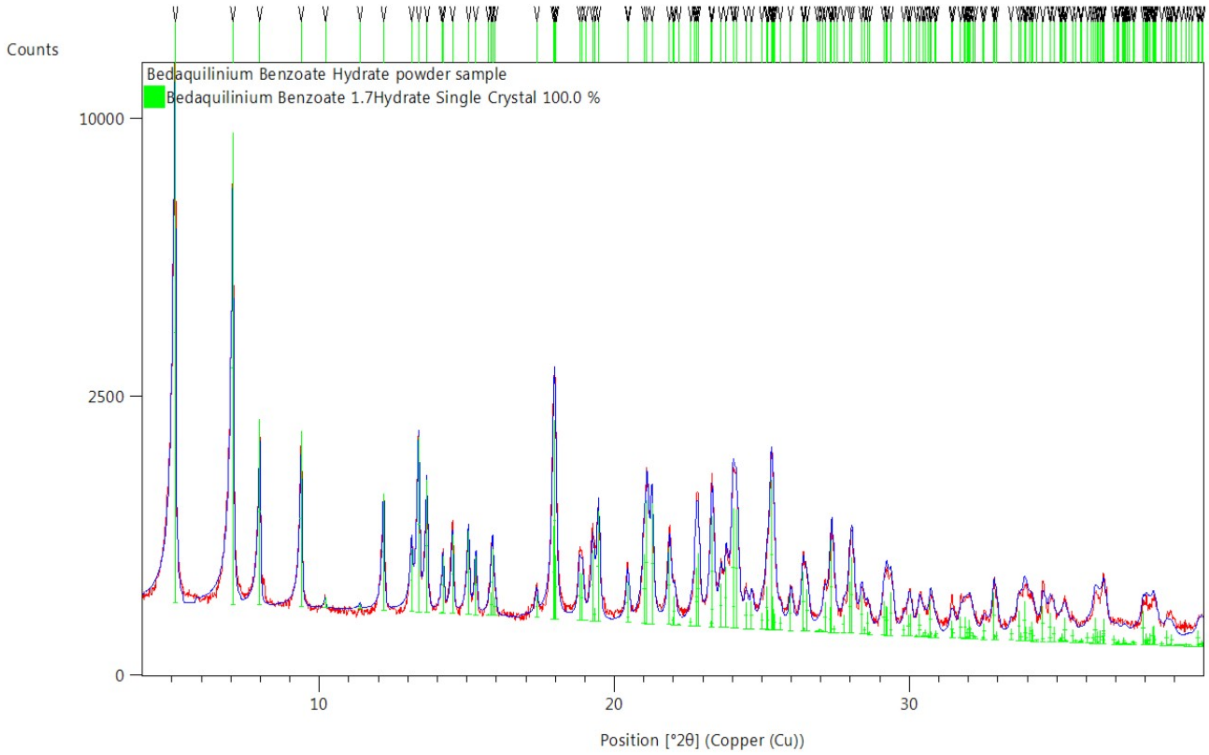

Figure S3. Powder XRD pattern (ambient temperature) of bedaquilinium benzoate powder obtained from acetone with Rietveld refinement fit against the single crystal structure of **4a**. The room temperature unit cell parameters refined to a = 12.7267(4), b = 8.0157(3), c = 17.6438(7) Å, beta = 99.7928(6)°, V = 1773.7(1) Å^3.

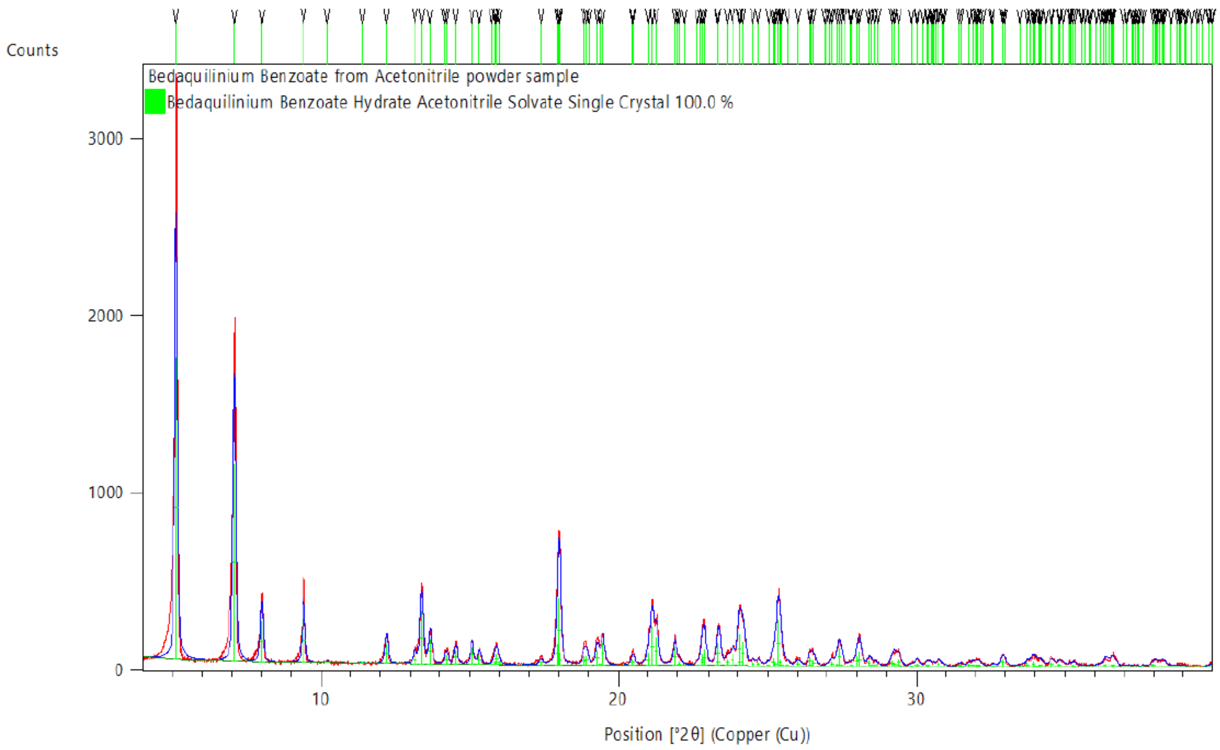

Figure S4. Powder XRD pattern (ambient temperature) of bedaquilinium benzoate powder obtained from acetonitrile with Rietveld refinement fit against the single crystal structure of **4b**. The room temperature unit cell parameters refined to a = 12.720(1), b = 8.0094(5), c = 17.626(2) Å, beta = 99.836(2)°, V = 1769.4(3) Å^3.

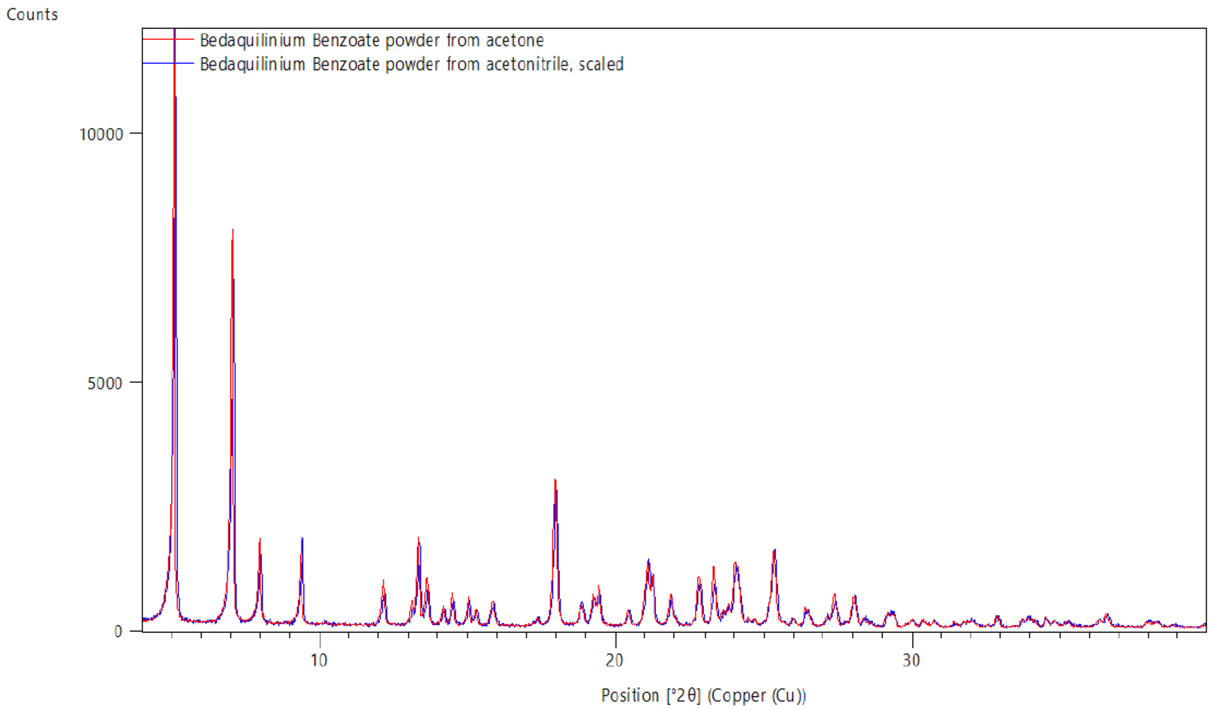

Figure S5. Overlay of experimental powder XRD patterns (ambient temperature) of bedaquilinium benzoate powder obtained from acetone and acetonitrile. The scale of the acetonitrile pattern was adjusted to fit that of the acetone pattern by matching the maxima of each pattern.

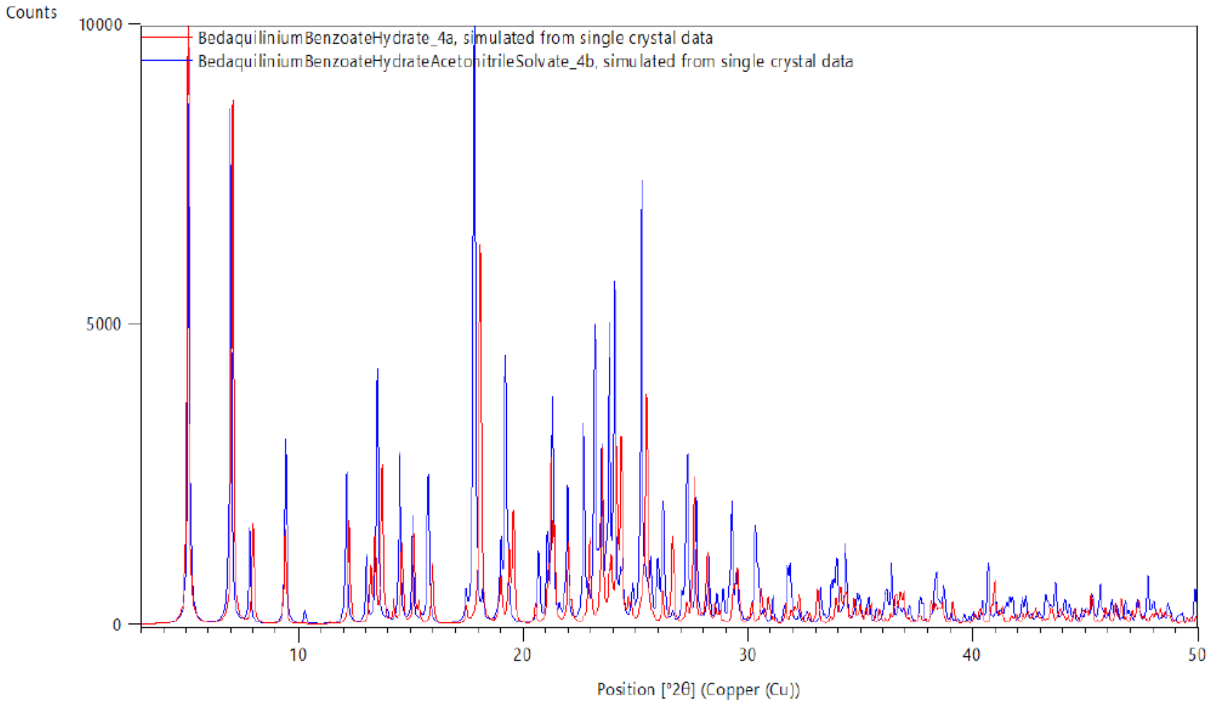

Figure S5. Overlay of powder XRD patterns of bedaquilinium benzoate **4a** obtained from acetone and **4b** from acetonitrile simulated from the single crystal data. Patterns created using Mercury (*Macrae et al.*, 2006).
